# Rapid response nursing triage outcomes for COVID-19: factors associated with patient’s participation in triage recommendations

**DOI:** 10.1186/s12911-023-02139-x

**Published:** 2023-03-08

**Authors:** Jyu-Lin Chen, Chen-Xi Lin, Mijung Park, Jerry John Nutor, Rosalind de Lisser, Thomas J. Hoffmann, Hannah J. Kim

**Affiliations:** 1 Department of Family Health Care Nursing, University of California, San Francisco, 2 Koret Way, San Francisco, CA 94143-0606 USA; 2 Davis Betty Irene Moore Hall, School of Nursing, University of California, 2570 48th St., Sacramento, CA 95817 USA; 3 Department of Epidemiology and Biostatistics, University of California, San Francisco, 513 Parnassus Ave, MSB, San Francisco, CA 94117 USA; 4 Kaiser Permanente Division of Research, 2000 Broadway, Oakland, CA 94612 USA

**Keywords:** COVID-19, Nursing triage, Patient participation, Symptom assessment

## Abstract

**Background:**

COVID-19 is an ongoing global health crisis with prevention and treatment recommendations rapidly changing. Rapid response telephone triage and advice services are critical in providing timely care during pandemics. Understanding patient participation with triage recommendations and factors associated with patient participation can assist in developing sensitive and timely interventions for receiving the treatment to prevent adverse health effects of COVID-19.

**Methods:**

This cohort study aimed to assess patient participation (percentage of patients who followed nursing triage suggestions from the COVID hotline) and identify factors associated with patient participation in four quarterly electronic health records from March 2020 to March 2021 (Phase 1: 14 March 2020–6 June 2020; Phase 2: 17 June 2020–16 September 2020; Phase 3: 17 September 2020–16 December 2020; Phase 4: 17 December 2020–16 March 2021). All callers who provided their symptoms (including asymptomatic with exposure to COVID) and received nursing triage were included in the study. Factors associated with patient participation were identified using multivariable logistic regression analyses, including demographic variables, comorbidity variables, health behaviors, and COVID-19-related symptoms.

**Results:**

The aggregated data included 9849 encounters/calls from 9021 unique participants. Results indicated: (1) 72.5% of patient participation rate; (2) participants advised to seek emergency department care had the lowest patient participation rate (43.4%); (3) patient participation was associated with older age, a lower comorbidity index, a lack of unexplained muscle aches, and respiratory symptoms. The absence of respiratory symptoms was the only factor significantly associated with patient participation in all four phases (OR = 0.75, 0.60, 0.64, 0.52, respectively). Older age was associated with higher patient participation in three out of four phases (OR = 1.01–1.02), and a lower Charlson comorbidity index was associated with higher patient participation in phase 3 and phase 4 (OR = 0.83, 0.88).

**Conclusion:**

Public participation in nursing triage during the COVID pandemic requires attention. This study supports using a nurse-led telehealth intervention and reveals crucial factors associated with patient participation. It highlighted the importance of timely follow-up in high-risk groups and the benefit of a telehealth intervention led by nurses serving as healthcare navigators during the COVID-19 pandemic.

## Background

The novel coronavirus (SARS-CoV-2; 2019-nCoV; COVID-19) is an urgent and ongoing global health crisis. As of 18 January 2022, California had about 6.8 million confirmed cases, with 77,306 deaths due to COVID-19 complications and a 21.1% test-positive rate [1]. To prepare for a sudden surge in patients needing critical medical care and to reduce the possibility of overwhelming health systems, healthcare providers must work together to manage the influx of potential patients at an early stage. This includes managing the influx at an early stage, and identifying patients in the community early through triage hotlines and clinics to provide appropriate and timely care for patients during the COVID-19 pandemic [2]. Nurses are uniquely positioned to lead community-based triage efforts to identify and recommend care for patients during the ongoing COVID-19 pandemic.

The outbreak of COVID-19 has impacted the usual face-to-face interactions between nurses and their patients. To provide high-quality clinical care and increase access to healthcare systems, many hospitals have increased the use of virtual and other telehealth systems to triage and care for patients. One of these technologies, Telephone Triage and Advice Services (TTAS), allows patients to speak to a nurse over the telephone to receive assessment and health advice [3]. Nurses can quickly obtain detailed travel and exposure histories and act as first responders for patients at elevated risk [4]. Automated screening algorithms may be built into the intake process, and local epidemiologic information can standardize screening and practice patterns across hospitals. Respiratory symptoms, which may be early signs of COVID-19, are most commonly evaluated using the TTAS approach.

Current research has revealed that TTAS are safe, promote care access, and increase service utilization [5–7]. During the ongoing pandemic, TTAS capabilities may have the potential to address increasing demands placed on overwhelmed health systems [7]. The COVID-19 pandemic has created and reinforced the opportunity and advantage for nurses and healthcare providers to provide assessment and recommendations via phone, especially when face-to-face interaction is limited and a large number of patients seek care recommendations. TTAS also enables access to care for people living in remote or underserved communities and those with underlying comorbid conditions with limited resources. COVID-19 is an ongoing public health challenge and mitigating public transmission with adherence to COVID-19 measures is needed. TTAS is run and led by nurses, serving as critical healthcare navigators who provide an essential function in the fight against COVID-19, offering patient education and triaging patients to the appropriate level of care. Patient participation is a patient’s subsequent action adherence to the health providers’ recommendation. Nevertheless, there is little evidence that documents the impact of a nurse-led COVID-19 rapid response telephone triage center on patient participation with the recommendations, and few studies have examined factors associated with patient participation with the COVID-19 triage recommendations [8, 9].

Understanding patient participation with triage recommendations and factors associated with patient participation (such as age, sex, health conditions, and symptoms) can assist in developing sensitive and timely interventions for receiving the treatment needed to prevent adverse health effects of COVID-19. Lessons revealed in this study can inform healthcare providers and policymakers in making recommendations for managing an early response to public health concerns.

## Methods

This study aimed to (a) assess patient participation rate with COVID-19 nursing triage recommendations in a large healthcare center in the United States and (b) identify factors (demographic data such as age, gender, comorbidity, obesity status, health behaviors such as smoking, drinking, and illicit drug used and symptoms) associated with patient participation with nurse recommendations at a University-Affiliated Medical Center. In this study, patient participation is defined as a patient’s subsequent action adherence to the telephone triage recommendation within 14 days of the initial triage call in the three-month phase.

A cohort study design was used to follow the patients over the two weeks after initiation of the triage hotline and advised them to follow the recommendations based on risk factors and reported symptoms. This study included patients from a large University-affiliated Medical Center in Northern California participating in the COVID-19 nursing triage hotline between 14 March 2020 and 21 March 2021. This study included a convenience sample of individuals who called the hotline, reported symptoms (including asymptomatic responses from patients who had exposure to COVID-19), and received nursing triage. Callers who called the hotline for COVID prevention or other COVID-related information without providing their symptom information for triage are omitted. This study utilized extensive data from the Medical Center’s electronic health records system. Study participants' consent was not required to use data from the electronic health records. This study has been approved by the Institutional Ethics Committee on Human Research of the University of California, San Francisco (20-31034).

A nurse triage hotline was established in a large University-affiliated medical center, Rapid Response Nursing Triage Hotline for COVID-19 called RN TO COVID, led by nurses who serve as health care navigators. Patients at this University-affiliated health center were advised to contact the COVID-19 hotline with questions or if they were experiencing COVID-19-related symptoms. All calls were taken by a triage nurse, and patients were asked about their symptoms, exposures, and comorbidities. The triage nurse then made care recommendations based on pre-determined triage protocols created by current clinical practice guidelines and COVID-19 symptomatology. Patients without symptoms but who had exposure to COVID-19 were asked to monitor their conditions and call back if they developed symptoms. Patients received four possible recommendations when they reported symptoms: self-care (SC), video acute care clinic visit (VACC within 24 h), respiratory symptom clinic (RSC in-person care within 12 h), and emergency department care (ED immediate care). A recent publication provides information on this triage protocol [10].

This study utilized extensive data from the Medical Center’s electronic health record system (Epic), available to hospital clinicians and researchers. Data were analyzed in four phases, based on quarterly periods of data collection from March 2020 to March 2021 (Phase 1: 14 March 2020–6 June 2020; Phase 2: 17 June 2020–16 September 2020; Phase 3: 17 September 2020–16 December 2020; Phase 4: 17 December 2020–16 March 2021). Individuals were included if they called the COVID-19 hotline, provided their symptoms, and received telephone triage decisions during the study period. This study used only initial call data per patient every three months. A response to the triage recommendation was determined by a patient’s action documented in the electronic medical record within 14 days of the triage call. Patients’ participation in the recommendation (VACC, RSC, and ED) were obtained in the electronic medical record system at the University-affiliated hospital system. All data extracted were de-identified. R v4.1.0 [11] and STATA SE v16.0 [12] were used for data cleaning and analysis.

### Measures

Dependent variable: patient participation in COVID-19 triage recommendation is defined as the patient’s subsequent action adherence to the telephone triage recommendation within 14 days of the initial triage call in the three-month phase. Independent variables include demographic characteristics (age, gender), comorbidity (Charlson comorbidity index and obesity status), health behaviors (smoking, drinking, and illicit drug use), and symptoms (fever, unexplained muscle aches, eye-nose-throat (ENT) symptoms, eye symptom-(i.e., eye redness and/or discharge, respiratory symptom, upper respiratory infection (URI) symptom, gastrointestinal (GI symptom), and altered mental status).

Charlson comorbidity index (CCI) is a weighted index as a continuous variable to predict the risk of death within one year of hospitalization for patients with specific comorbid conditions. Higher scores indicate a more severe condition and a poorer prognosis and have been used consistently in the literature [13]. Body Mass Index (BMI) was defined as the body mass (kg) divided by the square of the height (m^2^) and was calculated using self-reported weight and height. We defined obesity as having a BMI equal to or above 30 [14]. This was a dichotomous variable. Health behaviors included smoking, drinking, and illicit drug use as binary variables (0 = never, 1 = quit or currently use). COVID-related symptoms included fever, unexplained muscle aches, ENT, eye symptoms, respiratory symptoms, URI symptoms, GI symptoms, and altered mental status, which were included in the hotline nursing triage (0 = No, 1 = Yes).

### Analysis

Descriptive analyses were used to describe demographic characteristics and patient participation. Chi-square tests were performed to examine differences in the prevalence of obesity, health behaviors, and COVID-19-related symptoms between patients who followed the triage recommendation and those who did not follow the recommendation. Multivariable logistic regression analyses were performed to determine the odds ratios (ORs) and 95% confidence intervals (CIs) between demographic variables (gender and age), comorbidity variables (CCI and BMI), health behaviors (smoking, drinking, and illicit drug used), and COVID-related symptoms and patient participation based on four different pandemic phases and all four phases. All statistical analyses were performed with STATA SE v16.0 with a significance value set at *p* < 0.05 and a Bonferroni corrected (for 15 tests) significance level of 0.0033 for logistic regression models.

## Results

The analysis included 9849 encounters/calls from 9021 unique participants (mean age = 45.4 years, SD = 21.1). Overall, about 39.5% of patients self-identified as male, ranging from 38.8% in phase 2 to 40.3% in phase 4. On average, 21.8% of patients were obese, ranging from 19.5% in phase 3 and 24% in phase 1. In terms of health behaviors, in the overall sample, 30.1%, 66.1%, and 23.8% of patients reported smoking, drinking, and using illicit drugs, respectively (Table 1). The most commonly reported symptoms included URI symptoms (37.6%), respiratory symptoms (30%), unexplained muscle aches (17.8%), and GI symptoms (13%, Table 1).

**Table 1:** Characteristics of participants

	Phase 1	Phase 2	Phase 3	Phase 4	Total
	(n = 1769)	(n = 2887)	(n = 3250)	(n = 1943)	(N = 9849)
*Demographic*					
Age, mean (SD)	49.67 (19.85)	46.33 (20.90)	43.12 (20.78)	44.04 (22.35)	45.42 (21.10)
*Sex*					
Male	709 (40.1%)	1123 (38.8%)	1274 (39.1%)	785 (40.3%)	3891 (39.5%)
Female	1059 (59.9%)	1770 (61.2%)	1978 (60.9%)	1163 (59.7%)	5970 (60.5%)
*Comorbidity*					
Charlson comorbidity index, mean (SD)	0.99 (1.37)	0.82 (1.35)	0.61 (1.14)	0.72 (1.24)	0.76 (1.27)
Obesity (Yes)	398 (24.0%)	580 (22.1%)	579 (19.5%)	409 (23.2%)	1966 (21.8%)
*Health behaviors (Yes/No)*					
Smoking (Yes)	563 (33.2%)	819 (30.1%)	849 (28.2%)	539 (30.3%)	2770 (30.1%)
Drinking (Yes)	965 (61.2%)	1623 (66.0%)	1933 (70.7%)	992 (63.2%)	5513 (66.1%)
Illicit drug use (Yes)	331 (21.4%)	574 (24.1%)	689 (25.8%)	344 (22.5%)	1938 (23.8%)
*Symptoms (Yes/No)*					
Fever (Yes)	267 (15.1%)	0 (0%)	0 (0%)	0 (0%)	267 (2.7%)
Unexplained muscle aches (Yes)	387 (21.9%)	632 (21.9%)	481 (14.8%)	257 (13.2%)	1757 (17.8%)
ENT (Yes)	99 (5.6%)	152 (5.3%)	120 (3.7%)	76 (3.9%)	447 (4.5%)
Eye symptom (Yes)	40 (2.3%)	72 (2.5%)	63 (1.9%)	35 (1.8%)	210 (2.1%)
Respirator symptom (Yes)	831 (47.0%)	840 (29.1%)	809 (24.9%)	474 (24.4%)	2954 (30.0%)
URI symptom (Yes)	804 (45.5%)	1192 (41.3%)	1128 (34.7%)	583 (30.0%)	3707 (37.6%)
GI symptom (Yes)	329 (18.6%)	403 (14.0%)	350 (10.8%)	196 (10.1%)	1278 (13.0%)
Altered mental (Yes)	5 (0.3%)	8 (0.3%)	5 (0.2%)	3 (0.2%)	21 (0.2%)

Phase 1: 14 March 2020–6 June 2020. Phase 2: 17 June 2020–16 September 2020. Phase 3: 17 September 2020–16 December 2020. Phase 4: 17 December 2020–16 March 2021

On average, of the total cohort, about 3.4% of patients (n = 331) were directed to the ED, 14.2% to RSC (n = 1401), 18.3% to Self-care (n = 1798), 21.1% to VACC (n = 2082), and 43% to self-monitor (n = 4238, Table 2).

**Table 2:** Call results/recommendation

Phase	ED (%)	RSC (%)	SELF CARE (%)	VACC (%)	Monitor (%)	Total
1	80 (5.5)	256 (14.5)	446 (25.2)	566 (32.0)	421 (23.8)	1769
2	95 (3.3)	544 (18.8)	617 (21.4)	514 (17.8)	1117 (38.7)	2887
3	100 (3.0)	377 (11.6)	485 (19.0)	618 (19.0)	1670 (51.4)	3250
4	56 (2.9)	224 (11.5)	256 (13.2)	377 (19.4)	1030 (53.1)	1943
Total	331 (3.4)	1401 (14.2)	1798 (12.3)	2082 (21.1)	4238 (43.0)	9849

*ED* Emergency department immediate care; *RSC* Respiratory symptom clinic in-person care within 12 h; *VACC* Video acuter care clinic visits within 24 h

The overall patient participation rate for the following triage recommendations was 72.5%; in the four phases, patient participation rates were 69%, 68.7%, 77.8%, and 72.5%, respectively. Significant differences were found among the four recommendations across the four study phases. Participants advised to seek ED care had the lowest patient participation (43.4%), in comparison with participants advised to seek care at a video acute care clinic (57.6%), a respiratory screening center (71.6%), or to administer self-care (67.7%) (Table 3).

**Table 3:** Patient participation in nurse recommendations

Recommendation	Patient participation (%)				
	Phase 1–4	Phase 1	Phase 2	Phase 3	Phase 4
ED	137 (41.4)	37 (46.2)	28 (29.5)	49 (49.0)	23 (41.1)
RSC	804 (57.4)	158 (61.7)	334 (61.4)	216 (57.3)	96 (42.9)
VACC	1403 (67.6)	360 (63.6)	357 (69.5)	436 (70.6)	250 (66.3)
SELF CARE	1290 (71.6)	335 (75.1)	325 (52.7)	412 (85.1)	218 (85.2)
SELF MONITER	3507 (82.7)	331 (78.6)	940 (84.2)	1414 (84.6)	822 (79.8)
All	7141 (72.5)	1221 (69.0)	1984 (68.7)	2527 (77.8)	1409 (72.5)
Pearson chi2	156.4220***	33.9584***	281.3123***	218.3143***	181.9150***

*ED* Emergency department immediate care; *RSC* Respiratory symptom clinic in-person care within 12 h; *VACC* Video acuter care clinic visits within 24 h

***p < 0.001

In all 4 phases of aggregated data, older age, a lower Charlson comorbidity index (CCI), and lack of unexplained muscle aches and respiratory symptoms were associated with higher patient participation (Pseudo R2 = − 0.02; *p* < 0.001; Table 4). The absence of respiratory symptoms was the only factor significantly associated with higher patient participation in all periods [OR = 0.75, 0.60, 0.64, 0.52; 95% CI = (0.57, 0.91), (0.56, 0.85), (0.45, 0.75), (0.39, 0.70), respectively]. Older age was associated with higher patient participation in three out of four phases [OR = 1.01–1.02; 95% CI = (1.01, 1.02)], and a lower Charlson comorbidity index (CCI) was associated with higher patient participation in phase 3 and phase 4 [OR = 0.83, 0.88; 95% CI = (0.76, 0.90), (0.80, 0.97)] (Table 4).

**Table 4:** Logistic Regression model for patient’s participation in phases 1–;

	All phases	Phase 1	Phase 2	Phase 3	Phase 4
Sex	1.05	1.06	1.00	1.23	0.98
	[0.94, 1.17]	[0.84, 1.34]	[0.82, 1.22]	[0.99, 1.52]	[0.76, 1.27]
Age	1.01***	1.02***	1.01***	1.01	1.01**
	[1.006, 1.01]	[1.01, 1.02]	[1.00, 1.02]	[1.00, 1.01]	[1.00, 1.02]
Charlson comorbidity index	0.92***	0.93	1.02	0.83***	0.88*
	[0.88, 0.96]	[0.85, 1.01]	[0.95, 1.10]	[0.76, 0.90]	[0.80, 0.97]
Obesity	1.01	1.05	1.24	1.00	1.05
	[0.95, 1.08]	[0.81, 1.35]	[0.99, 1.55]	[0.96, 1.04]	[0.79, 1.39]
Smoking	1.10	1.07	1.23	0.96	1.08
	[0.97, 1.23]	[0.83, 1.39]	[1.00, 1.52]	[0.77, 1.21]	[0.82, 1.43]
Drinking	0.94	0.89	0.98	0.91	0.93
	[0.84, 1.05]	[0.70, 1.13]	[0.80, 1.21]	[0.73, 1.14]	[0.71, 1.21]
Drug used	1.09	1.26	1.14	0.91	1.09
	[0.96, 1.25]	[0.93, 1.70]	[0.90, 1.44]	[0.71, 1.15]	[0.80, 1.50]
Fever	0.79	0.85	1.00	1.00	1.00
	[0.60, 1.04]	[0.63, 1.14]	[1.00, 1.00]	[1.00, 1.00]	[1.00, 1.00]
Unexplained muscle aches	0.82**	0.93	0.84	0.65**	1.01
	[0.75, 0.52]	[0.69, 1.26]	[0.67, 1.05]	[0.49, 0.86]	[0.69, 1.49]
ENT symptom	1.16	0.86	1.34	1.21	1.08
	[0.91,1.47]	[0.53, 1.39]	[0.89, 2.02]	[0.73, 2.02]	[0.59, 1.95]
Eye symptom	0.83	0.71	0.92	0.72	1.02
	[0.60, 1.15]	[0.35, 1.45]	[0.54, 1.57]	[0.38, 1.34]	[0.42, 2.52]
Respiratory symptom	0.60***	0.72**	0.69**	0.58***	0.52***
	[0.54, 0.67]	[0.57, 0.91]	[0.56, 0.85]	[0.45, 0.75]	[0.39, 0.70]
URI symptom	0.93	1.24	0.65***	1.27	1.03
	[0.82, 1.05]	[0.95, 1.62]	[0.53, 0.80]	[0.98, 1.65]	[0.76, 1.40]
GI symptom	0.89	0.98	0.97	0.86	0.77
	[0.77, 1.04]	[0.73, 1.32]	[0.74, 1.26]	[0.63, 1.18]	[0.51, 1.15]
Altered mental	0.74	1.00	1.24	0.37	1.00
	[0.27, 2.05]	[1.00, 1.00]	[0.24, 6.41]	[0.05, 2.88]	[1.00, 1.00]
Observations	7669	1484	2250	2497	1433

Exponentiated coefficients; 95% confidence intervals in brackets

*p < 0.05, **p < 0.01, ***p < 0.001

## Discussion

This study aimed to assess the utilization of the COVID-19 hotline and patient participation with COVID-19 nursing triage recommendations and identify factors associated with patient participation in the early COVID-19 pandemic. More than 9,800 patients called the nurse-led triage COVID hotline during the first 12 months of the pandemic. The top three reported symptoms reported by patients include URI symptoms (37.6%), respiratory symptoms (30%), and unexplained muscle aches (17.8%). Of patients who reported symptoms, 21.1% were advised to VACC, 18.3% to Self-care, 14.2% to RSC, and 3.4% of patients to ED. In this study, the average patient participation rate of nursing triage recommendations was 72.5%, ranging from 68.7 to 77.8%. We found that participants advised to seek ED care had the lowest patient participation (43.4%). The absence of respiratory symptoms, older age, and lower Charlson comorbidity index are associated with higher patient participation in the recommendations.

The triage nurses’ recommendations were based on the triage protocol, including symptoms and other high-risk factors (such as age and other chronic health conditions). The COVID-19 hotline was heavily utilized by people seeking nursing triage and recommendations in the community. Our study found that about one-third of patients reported URI or respiratory symptoms. These respiratory-related symptoms reported by patients are consistent with studies suggesting that COVID-19 primarily targets the respiratory system. The virus can enter the respiratory tract through the mucous membranes of the mouth, nose, and eyes [15–17]. A recent systematic review and meta-analysis study on the prevalence of symptoms in adults from nine countries found that the most common clinical presentation of severe COVID-19 was fever (78%), followed by cough (57%) and fatigue (31%) [15]. Current evidence suggests that individuals infected with COVID-19 experience various symptoms, from mild to severe conditions, and symptoms typically occur 2–14 days after virus exposure [15]. The time gap between exposure to COVID and the onset of symptoms may be responsible for the low prevalence of some symptoms in our study since some callers reached the hotline after being exposed to COVID but without symptoms. As the virus mutates and evolves, the continued assessment of the symptoms reported by people with COVID-19 is critical in understanding the symptom presentation and influencing how patients perceive the need to follow the nurses’ recommendations and receive treatment when necessary. The average patient participation rate in nursing triage recommendations was 72.5%, ranging from 68.7 to 77.8%. Patients advised ED care had the lowest patient participation rate (43.4%), which is very concerning as the progression of severe symptoms can occur quickly and increase the risk for further health deterioration and delayed necessary hospitalization. Our findings are consistent with national data indicating that ED visits were lower between April and June 2020 [18]. Delayed ED care increases the risk of not receiving appropriate care and not getting an accurate diagnosis, and prolonged hospitalization increases mortality [19, 20]. A current study in Sweden found the overall 60-day mortality rate as 17.4% during the first year of the pandemic [20]. Studies have found high mortality rates ranging from 35 to 62% for critically ill patients with coronavirus [21–23]. Some possible rationales for non-adherence to the recommendation to ED care could include that individuals were concerned about the long waiting time in the ED or the likelihood of being infected with COVID while staying in the ED. Additionally, during the stay-at-home order, individuals may have had limited assistance at home, thus impacting their ability to seek acute ED care, and others may have been concerned about the cost of ED care. Timely treatment is essential in reducing the mortality associated with COVID-19. Future studies will need to explore the individual perception of the severity of illness and individuals’ rationale for not going to ED when advised.

Our study found that common factors associated with patient participation in the triage recommendation include the absence of respiratory symptoms and older and lower comorbidity. As the most common symptoms reported by people with COVID-19 include fever and dry cough [17], individuals without respiratory and lower comorbidity may be more likely to be asked to perform self-care or VACC, which is easier to follow through. Consistent with previous studies, individuals with a medical history and old age have an increased risk for infection and poorer COVID-19 outcomes due to weaker immune systems [24–26]. In the early pandemic, although there was limited knowledge of COVID-19 and its impact on health, there were several public health campaigns and regular announcements related to this newly discovered virus on media. These public announcements discussed the incidence of COVID-19 and shared information on the high-risk group for COVID-19 infection and mortality (such as older age and individuals with existing health conditions). The media coverage of COVID-19 influence individuals’ perception of their risk and decision on behavior changes (i.e., wearing a face mask, keeping social distance, frequent handwashing, getting medical attention) and vaccination intention [27, 28]. As older age has been reported to increase the risk for COVID-19-related hospitalization and mortality, individuals with older age may be more aware of their risk for COVID-19 and the health effects of COVID-19; thus, they are more likely to follow the nurses’ recommendations. To increase patient participation in nurses’ recommendations, public health organizations may need to engage various communities in health literacy champions that are culturally and linguistically appropriate to increase the understanding of the current recommendations for public health issues. Future studies may also need to explore the role of media on COVID-19 behavior change and medical care-seeking behaviors.

The results of this study provide recommendations for healthcare providers and policymakers regarding the management of the ongoing COVID-19 response and future public health emergencies. First, the utilization and participation rate of the nurse-based telephone triage system was moderate to high. A nurse-based telephone triage system based on the most up-to-date evidence can be quickly established to respond to emergency health events. The timeliness of the response is feasible and critical in providing timely and appropriate care. Second, timely follow-up with the patient regarding the recommendations, understanding the patient’s hesitancy in following the recommendation, and adapting policy in real-time are important factors in addressing the ongoing COVID-19 pandemic, as well as improving patient health outcomes. Third, patients advised to seek emergency department care had the lowest patient participation (43.4%), revealing the importance of timely follow-up for high-risk groups. Future clinical pathways or practice guidelines may need to consider factors (respiratory symptoms, younger age, and higher Charlson comorbidity index) in clinical decision-making.

This study is one of the first to explore the utilization of the nurse-led triage COVID-19 hotline. The results of the study should be interpreted with caution. Some limitations of the study include convenient sampling, the use of only electronic medical record data within a university-affiliated hospital, and self-reported data. The fewer report of some symptoms, such as altered mental conditions and fevers, may cause the models to be unstable, and the results must be interpreted carefully. Moreover, the percentage of variance explained by the model and ORs for age is small and other variables such as living alone or with family, history of illness, and previous relationships with healthcare workers could be included in future analyses. Another limitation is that we were unable to truly validate whether patients have performed self-care. We assumed that patients who did not use other services (such as VCC, RSC, and ED) within 14 days performed self-care. Future studies may reach out to patients concerning their self-care behaviors.

Future studies may need to consider other potential factors (such as symptom severity, medical history, knowledge about COVID-19, exposure to COVID-19, social determinants of health variables, access to treatment, and social support) related to patient participation in recommendations made by nurses and matching groups. Future work may have broader generalizability if other settings were included and a mixed-method design was used to examine barriers and facilitators following triage recommendations.

## Conclusion

The average patient participation rate was 72.5%, with the ED being the lowest rate. The absence of respiratory symptoms, older age, and lower Charlson comorbidity index are associated with higher patient participation. Public participation in nursing triage during the COVID pandemic requires attention. This study supports using a nurse-led telehealth intervention, where nurses serve as critical healthcare navigators, providing assessment and care recommendations during the COVID-19 pandemic. While many adhered to nurse triage recommendations, those determined to be most critical did not; this is a concerning finding that warrants future study.

## Data Availability

The data presented in this study are available on request from the corresponding author. The data are not publicly available due to them containing personal information.

## Abbreviations

TTAS: Telephone triage and advice services
OR: Odds ratio
SC: Self-care
VACC: Video acute care clinic visit
RSC: Respiratory symptom clinic (RSC in-person care within 12 h) and emergency
ED: Emergency department care
ENT: Eye-nose-throat (ENT) symptoms
URI: Upper respiratory infection
GI: Gastrointestinal
CCI: Charlson comorbidity index
BMI: Body mass index (BMI)
CI: Confidence interval
SD: Standard deviation
